# Supplementary material for: Causal Relationship between Aging and Anorexia Nervosa: A White-Matter-Microstructure-Mediated Mendelian Randomization Analysis
Source: Biomedicines. 2024 Aug 16;12(8):1874. doi: 10.3390/biomedicines12081874 (PMC11351342; doi:10.3390/biomedicines12081874)
Supplement: Supplementary file 1 [file biomedicines-12-01874-s001.zip › Supplementary Figure/Figure S8/Leaveoneout plot of MR analysis for TL to ubm-b-2023.pdf]

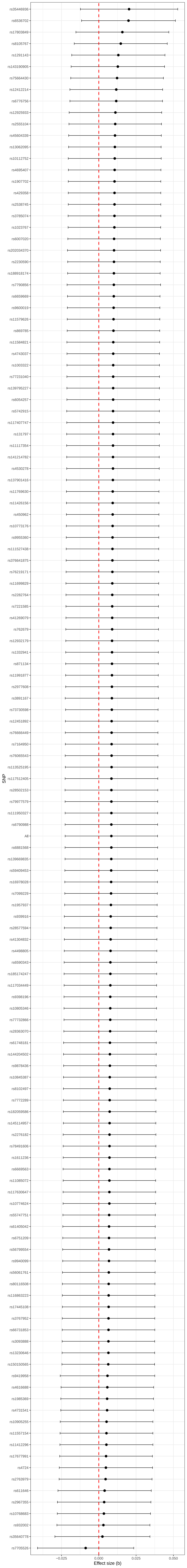
rs35446936
rs6536702
rs17803849
rs8105767
rs1291143
rs143190905
rs75664430
rs12412214
rs6776756
rs12925933
rs2555104
rs45604339
rs13062095
rs10112752
rs4695407
rs1907702
rs429358
rs2538745
rs3785074
rs1023767
rs6007020
rs202034370
rs2230590
rs188918174
rs7790856
rs6659669
rs9600019
rs11579626
rs869785
rs11584821
rs4743037
rs1003322
rs77231040
rs139795227
rs6054257
rs5742915
rs117407747
rs131797
rs11117354
rs141214782
rs4530278
rs137901416
rs11769630
rs11426156
rs450962
rs10773176
rs9955360
rs111527438
rs376641875
rs76219171
rs11699829
rs2282764
rs7221585
rs41269079
rs762679
rs12932179
rs1332941
rs871134
rs11991877
rs2977608
rs3891167
rs73730598
rs12451892
rs76666449
rs7164950
rs76065543
rs113525195
rs117512405
rs28502163
rs79977579
rs111950227
rs6790988
All
rs6881568
rs139669835
rs59409453
rs16978028
rs7099229
rs1957937
rs939916
rs28577594
rs41304832
rs4498805
rs6590343
rs185174247
rs117034449
rs9398196
rs10805346
rs77732866
rs28363070
rs61748181
rs144204502
rs9878436
rs10845387
rs8102497
rs7772289
rs182059586
rs145114957
rs2276182
rs78491606
rs1611236
rs6669563
rs11085072
rs117630647
rs10774624
rs55747751
rs61405042
rs6751209
rs56799554
rs9940099
rs56061761
rs80116508
rs116863223
rs17445108
rs3767952
rs66731853
rs3093888
rs13230646
rs150150565
rs9419958
rs4616688
rs1985369
rs4731541
rs109905255
rs11557154
rs11412296
rs17677991
rs4724
rs2763979
rs611646
rs2967355
rs10768683
rs932002
rs35640778
rs7705526
SNP
-0.025
0.000
0.025
0.050
Effect size (b)
